# Multimodal Ophthalmic Imaging in Spinocerebellar Ataxia Type 7

**DOI:** 10.3390/life13112169

**Published:** 2023-11-06

**Authors:** Chiara Ciancimino, Mariachiara Di Pippo, Gregorio Antonio Manco, Silvia Romano, Giovanni Ristori, Gianluca Scuderi, Solmaz Abdolrahimzadeh

**Affiliations:** 1 Ophthalmology Unit, Department of Neurosciences, Mental Health and Sensory Organs (NESMOS), St. Andrea Hospital, “Sapienza” University of Rome, Via di Grottarossa 1035/1039, 00189 Rome, Italy; chiara.ciancimino@uniroma1.it (C.C.); mariachiara.dipippo@uniroma1.it (M.D.P.); gregorio.manco@uniroma1.it (G.A.M.); solmaz.abdolrahimzadeh@uniroma1.it (S.A.); 2 Center for Experimental Neurological Therapies (CENTERS), Department of Neurosciences, Mental Health and Sensory Organs (NESMOS), St. Andrea Hospital, “Sapienza” University of Rome, Via di Grottarossa 1035/1039, 00189 Rome, Italy; silvia.romano@uniroma1.it (S.R.); giovanni.ristori@uniroma1.it (G.R.); 3 Neuroimmunology Unit, IRCCS Fondazione Santa Lucia, Via Ardeatina, 306/354, 00179 Rome, Italy

**Keywords:** spinocerebellar ataxia, SCA7, ocular manifestations, color fundus photography, spectral domain optical coherence tomography, multimodal imaging, near infrared reflectance, electrophysiological tests

## Abstract

The aim of this case series and narrative literature review is to highlight the importance of multimodal imaging in the ophthalmological examination of patients with spinocerebellar ataxia type 7 and provide a summary of the most relevant imaging techniques. Three patients with SCA7 were included in this case series. A literature review revealed twenty-one publications regarding ocular manifestations of SCA7, and the most relevant aspects are summarized. The role of different imaging techniques in the follow-up of SCA7 patients is analyzed, including color vision testing, corneal endothelial topography, color fundus photography (CFP) and autofluorescence, near infrared reflectance imaging, spectral domain optical coherence tomography (SDOCT), visual field examination, and electrophysiological tests. SDOCT provides a rapid and non-invasive imaging evaluation of disease progression over time. Additional examination including NIR imaging can provide further information on photoreceptor alteration and subtle disruption of the RPE, which are not evident with CFP at an early stage. Electrophysiological tests provide essential results on the state of cone and rod dystrophy, which could be paramount in guiding future genetic therapies. Multimodal imaging is a valuable addition to comprehensive ophthalmological examination in the diagnosis and management of patients with SCA7.

## 1. Introduction

Spinocerebellar ataxias (SCAs) are genetic neurodegenerative diseases characterized by progressive loss of balance and coordination and often associated with additional symptoms such as slurred speech or progressive visual loss. Different subtypes are present within the SCA group, including SCA1, which is the most common with a prevalence of 1–5 per 1,000,000 individuals, SCA2, SCA3, SCA6, SCA7, and SCA17. Currently, 27 distinct SCA forms have been recognized, and more are added yearly. The genetic mutations associated with these can be grouped into three classes: CAG/polyglutamine (poly Q) ataxias with expansion; ataxias due to non-protein coding repeat expansions; and ataxias resulting from standard mutations. A genetic expansion in a CAG repeat sequence that translates into a poly Q tract is the pathophysiological basis for SCA7 [1,2]. 

This review and case series focuses on SCA7, an autosomal dominant neurodegenerative disorder with a reported incidence of <1 per 100,000 individuals. It seems to be the only form within the SCA family to present a decrease in visual acuity prevalently due to cone and rod photoreceptor dystrophy [3].

Genetic studies have identified *ATXN7*, a gene found on chromosome 3, to be affected in SCA7. *ATXN7* alleles typically have 4–35 CAG repeats, with most carrying 10 CAG repeats. SCA7 patients have mutant alleles with 36 or more CAG repeats and can reach more than 460 repeats [2,4,5]. A common feature in polyQ diseases is the intracellular accumulation of amyloid-like aggregates containing protein fragments bearing the polyQ expansion. In SCA7, large aggregates in cell nuclei are observed in numerous brain tissues; these are known as mutant ATXN7 (mATXN7) aggregates and are observed as nuclear inclusions (NI) by immunohistochemistry [6]. As in other polyQ diseases, such as Huntington’s disease, SCA7 patients show an inverse relationship between the number of CAG repeats and the age of disease presentation, whereas there is a direct relationship with the severity of neurological and ophthalmological symptoms [7]. Furthermore, successive generations present disease anticipation, mainly due to paternal transmission. On average, symptoms begin around 30 years of age; however, there is variability, with reported onset ranging from 1 month to 76 years [8]. CAG repeats <59 are more commonly associated with adult onset (age >30 years) with mainly cerebellar and ataxic findings. Repeats over 59 are typically associated with adolescent or young adult onset, and visual impairment is an early symptom. Juvenile and more aggressive forms have been diagnosed in patients with CAG expansion sizes between 60–100, and infantile forms, incompatible with life and associated with multiorgan failure, have been reported with repeat sizes ranging between 200–400 [7,9].

*ATXN7* is a core component of SAGA complexes (Spt-Ada-Gcn5 Acetyltransferase) involved in chromatin remodeling. In particular, the C-terminus of *ATXN7* extends into SAGA, interacting with proteins in the core module. SAGA is a large complex best described as a chromatin-modifying transcriptional coactivator complex which acetylates and deubiquitinates histones preparing for transcriptional activation [10]. The core module assists with pre-initiation complex assembly, while the splicing module assists with gene activation and transcript splicing. 

Transcriptome analysis of SCA7 mouse retina models revealed an early and progressive downregulation of most photoreceptor-specific genes leading to a progressive reduction of electroretinography activity and retinal thinning. The expression profile of SCA7 in mouse cerebellum models showed the downregulation of genes involved in the maintenance and function of neuronal dendrites and myelin sheath. The postmortem cerebellum of SCA7 patients reveals neuronal loss in the Purkinje cell layer, dentate nuclei, and granule cell layer [2,6]. Mitochondrial activity has been analyzed in SCA7, and the results show that it is abnormal in mouse retina and presents a decreased electron transport chain activity and metabolic acidosis in muscle tissue [11]. In SCA7 knock-in and transgenic mouse models, mATXN7 accumulates faster in the nuclei of vulnerable neurons such as photoreceptors and Purkinje cells. mATXN7 nuclear inclusions also disrupt many cellular proteins and activities, thus contributing to pathogenesis. Proteasomes, chaperones, RNA binding proteins, transcription activators such as the CREB-binding protein, and subunits of the SAGA complex may therefore lose their biological functions [2].

As SCA7 is a rare disease, it is challenging to make an early diagnosis; however, counseling families is fundamental due to the autosomal dominant transmission and the impressive anticipation phenomenon. 

In this case series and literature review, the authors describe three patients with ophthalmological signs of SCA7 and summarize available case reports on SCA7, documenting the ophthalmologic examinations and imaging techniques performed for each patient to support other doctors in the early detection and follow-up of this complex neurodegenerative and ocular pathology. 

## 2. Materials and Methods

### 2.1. Case Series

Three patients with confirmed genetic diagnosis of SCA7 followed at the neurology department of the University of Rome Sapienza, Sant’ Andrea Hospital, were included in this case series. The patients were informed of the study and signed informed consent. The study was carried out according to the tenets of the Declaration of Helsinki. The patients underwent a complete neurological and ophthalmological assessment with multimodal imaging. 

### 2.2. Neurological Examination

The scale for assessing and rating cerebellar ataxia (SARA) was evaluated in all patients; it assesses, in a semiquantitative way, the general neurological burden in addition to ataxia. Specifically, it evaluates eight items for a total score of 0 (no ataxia) to 40 (most severe ataxia) [12].

### 2.3. Ophthalmological Examination

A comprehensive ophthalmologic evaluation including the best corrected visual acuity (BCVA), the Farnsworth-Munsell Dichotomous D-15 vision test, and slit lamp anterior segment biomicroscopy with applanation tonometry and fundus examination was conducted. Color fundus photography (CFP) and spectral domain optical coherence tomography (SDOCT) were both taken with a Solix (Optovue, Fremont, CA, USA) device. A 30:2 Humphrey visual field examination was performed when available based on the patient’s possibilities. For patient 1, further examinations, including electroretinograms (ERG) and visually evoked potentials (VEP), were performed, and for patient 3, near-infrared reflectance imaging (NIR) and fundus autofluorescence (FAF) were also included, utilizing a Heidelberg Spectralis HRA (Heidelberg, Germany) device. 

### 2.4. Literature Review

A literature search of the MEDLINE database was performed using the terms “spinocerebellar ataxia type 7” case report” or “case series,” “SCA7 ocular manifestations,” and “SCA7 ophthalmic manifestations” and “SCA7” for articles in English accessed through August 2023. The articles were selected if they reported and described ophthalmologic procedures for diagnosing and following patients with a known genetic mutation causing SCA7. Twenty-one manuscripts were selected and included in this narrative literature review, as they were case reports or more extensive case series that reported prevalently ocular manifestations and ophthalmic imaging characteristics of SCA7. Reference lists of the selected manuscripts were also analyzed to retrieve other relevant studies. 

## 3. Case Series

### 3.1. Case 1

**Figure 1:**
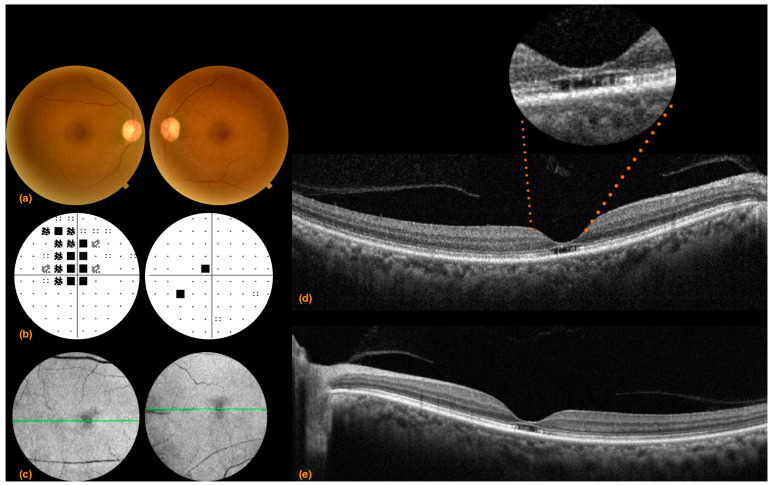
Case 1: (**a**) Right and left eye color fundus photography showing a slightly pale optic nerve head, absence of foveal reflex, mottled macular pigmentation, and arteriolar thinning; (**b**) right and left eye 30:2 Humphrey visual field pattern deviations. A bilateral central scotoma that is more advanced and extends to the superior hemifield in the right compared to the left eye is evident; (**c**) left eye spectral domain optical coherence tomography 30 μm en-face infrared reflectance showing retinal pigment epithelium hyporeflectivity (**d**) right eye and (**e**) left eye spectral domain optical coherence tomography showed central retinal thinning, abnormalities in the outer retinal layers, loss of ellipsoid zone, and disruption of the foveal photoreceptors in the right eye more than the left eye.

**Figure 2:**
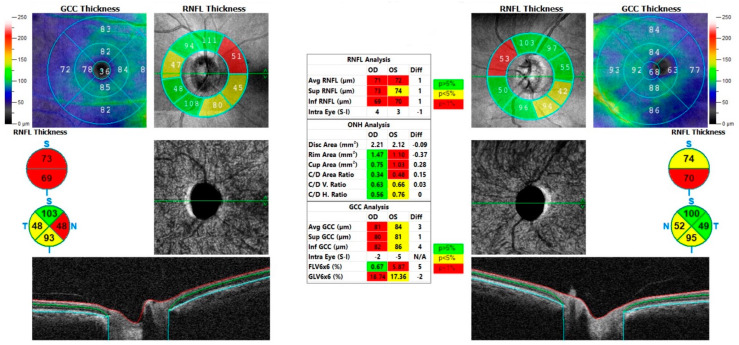
Case 1. Retinal nerve fiber layer (RNFL) and ganglion cell complex (GCC) analysis of right and left optic nerves. Both layers are thinner; nasal RNFL thickness is more reduced compared to temporal thickness.

**Figure 3:**
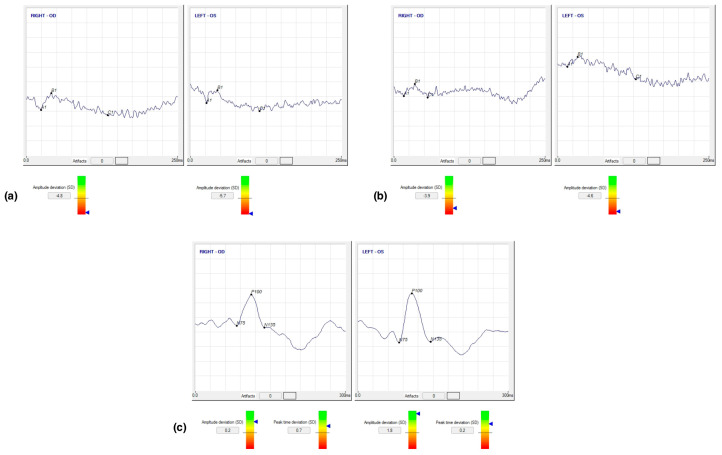
Case 1. (**a**) Scotopic and (**b**) photopic electroretinogram responses demonstrating reduced amplitude, (**c**) visual evoked potentials demonstrating a normal amplitude and peak time deviation in both eyes.

A 64-year-old Congolese woman presented with progressive gait ataxia and mild bilateral visual loss. DNA analysis revealed an increased CAG repeat number of 46 in one *ATXN7* allele, confirming the diagnosis of SCA7. The neurological examination revealed a SARA score of 14. The BCVA scores were 42 and 43 early treatment diabetic retinopathy study (ETDRS) letters in the right and left eyes, respectively, both corresponding to the 0.2 LogMAR equivalent. The Farnsworth-Munsell Dichotomous D-15 binocular vision test demonstrated blue-yellow color blindness, and the anterior segment examination was unremarkable, while fundoscopy and CFP showed pale optic discs, attenuated retinal arteries with pigment mottling at the macula (Figure 1a). Central scotomas were present at 30:2 Humphrey visual field examination (Figure 1b). SDOCT showed central retinal thinning, abnormalities in the outer retinal layers, loss of ellipsoid zone, and disruption of the foveal photoreceptors in the right eye more than the left eye (Figure 1d,e). En-face infrared reflectance images of the retinal pigment epithelium (RPE) showed central hyporeflectivity (Figure 1c). Central retinal thicknesses were 148 μm and 166 μm in the right and left eyes, respectively, parameters that remained steady at follow-up 6 months from baseline examination. Retinal nerve fiber layer (RNFL) and macular ganglion cell complex (GCC) thinning occurred in both eyes. RNFL thickness was better preserved in the temporal with respect to the nasal fibers (Figure 2). VEP were within limits in amplitude and peak time, while ERG showed a reduced cone response with a slightly inferior decrease in rod response (Figure 3).

### 3.2. Case 2

**Figure 4:**
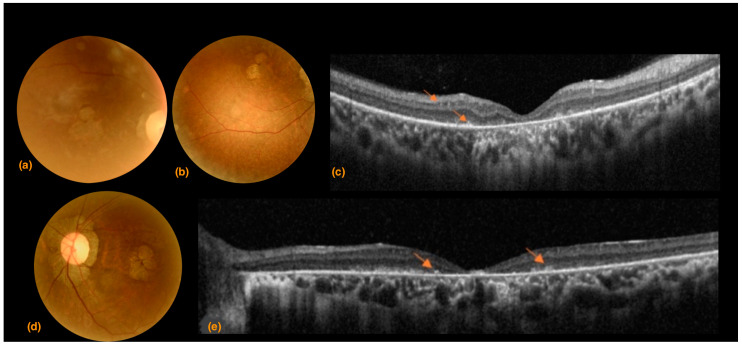
Case 2. (**a**) Superior right eye hemiretina, (**b**) inferior right eye hemiretina, and (**d**) left eye color fundus photography. A pale optic nerve, straitening and thinning of retinal arteries, extensive peripapillary and macular atrophy associated with tessellation and increased visibility of the underlying choroid; (**c**) right eye and (**e**) left eye spectral domain optical coherence tomography B scans. Both eyes present retinal thinning with increased signal backscattering and increased foveal depression.; (**c**) focal hyperreflectivity of the internal limiting membrane and disorganized external retinal layers, especially in the foveal and perifoveal area; arrows indicate hyperreflective dots present in different retinal layer (**e**) focal hyperreflectivity of the internal limiting membrane, thinning and morphological alteration of the external retinal layers with distinctive hyperreflective dots/humps (arrows), and extensive interruptions.

A 30-year-old Israeli woman presented with strongly impaired mobility and profound visual loss. The patient came to Sant’ Andrea Hospital with a positive genetic analysis for SCA7 without specification of the number of CAG repeats. Her SARA score was 23, indicating a strongly compromised gait. The BCVA results were counts fingers at 60 cm in the right eye and hand motion in the left eye. Eye motility was limited bilaterally in all directions of gaze, and the patient presented intense photophobia. Color vision and visual field examination were not possible due to the low visual acuity of the patient. Fundoscopic examination and photography presented optic nerve pallor with peripapillary atrophy, vascular attenuation, and retinal atrophy (Figure 4a,b,d). SDOCT imaging showed diffuse increased backscattering and significantly reduced central retinal thicknesses of 52 μm and 41 μm in the right and left eyes, respectively, indicating diffuse retinal atrophy. Thinning occurred mainly in the external retinal layers with altered morphology and reflectivity, indicating extensive atrophy throughout the entire SDOCT b scan (Figure 4c,e).

### 3.3. Case 3

**Figure 5:**
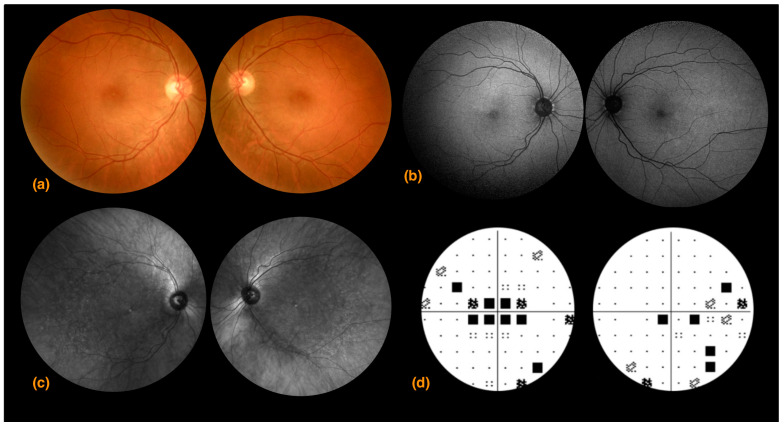
Case 3. (**a**) Right and left eye color fundus photography (CFP) shows a normal-appearing retina with loss of foveal reflex and tessellation with choroidal vascularization becoming evident along the inferior vascular arcade; (**b**) right eye and left eye fundus autofluorescence shows a normal appearing macular hypoautofluorescence; (**c**) right and left eye near-infrared reflectance shows small hyperreflective dots at both foveae and a diffuse granular appearance of the posterior pole, indicating areas of pigment epithelium irregularities, not evident on CFP; (**d**) right and left eye 30:2 Humphrey visual field pattern deviation shows a central scotoma in the right eye and central and nasal defects in the left eye.

**Figure 6:**
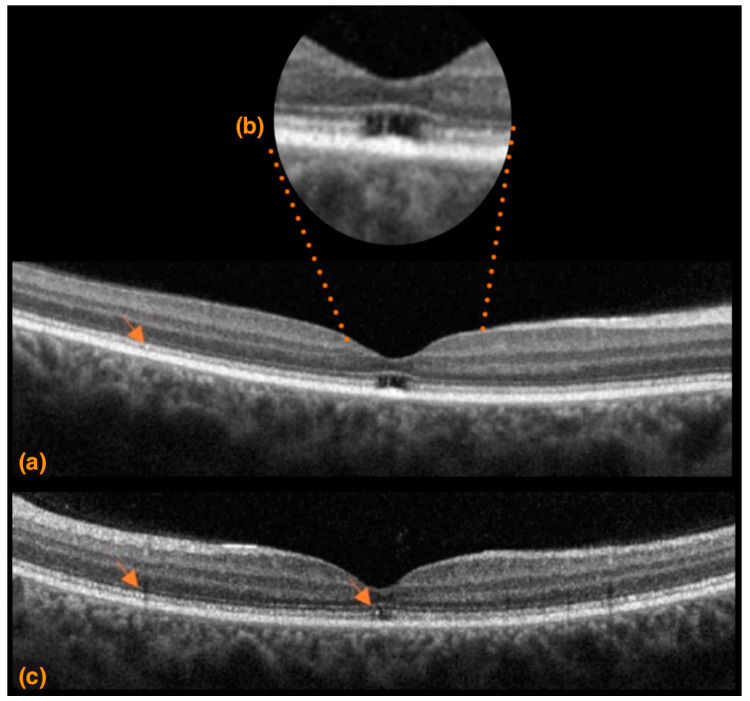
Case 3 (**a**,**b**) right eye and (**c**) left eye spectral domain optical coherence tomography B scan showing slightly steep foveal depression, focal loss of the ellipsoid zone in the right eye > left eye, and generalized granular appearance of the external retinal layers with some hyperreflective dots (arrows).

**Figure 7:**
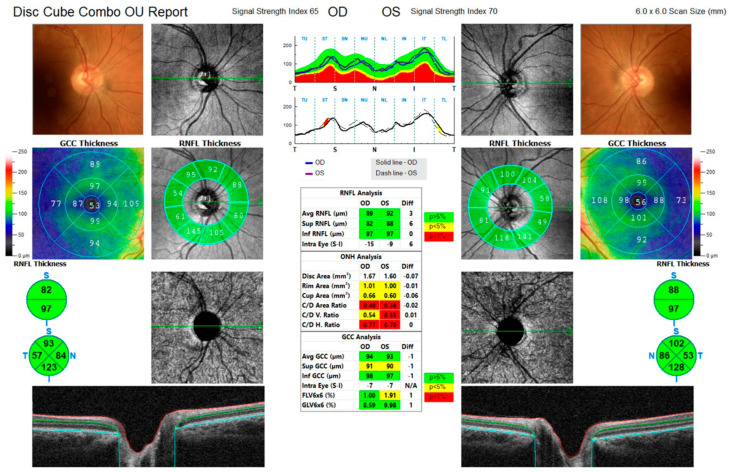
Case 3. Optic nerve analysis. Retinal nerve fiber layer and macular ganglion cell complex thickness were normal in both eyes.

A 32-year-old Italian woman presented with compromised general motility and progressive bilateral visual loss. Genetic analysis of *ATXN7* demonstrated 44 CAG repeats. The BCVA values were 4 and 9 ETDRS in the right and left eyes, respectively, corresponding to 1.0 and 0.9 LogMAR equivalents. Farnsworth-Munsell Dichotomous D-15 binocular vision test demonstrated red-green color blindness. Fundoscopic examination and photography showed a healthy optic nerve, absence of foveal reflex, and slight foveal hypopigmentation (Figure 5a). FAF showed a normal appearing macular hypoautofluorescence (Figure 5b). Upon SDOCT scanning, retinal thinning became apparent, with central retinal thicknesses of 188 μm and 190 μm in the right and left eyes, respectively. The foveal depression and the morphology and reflectivity of the internal retinal layers were preserved. In the foveal area, there was a focal loss of the ellipsoid zone in the right eye more so than in the left eye, but the morphology of the external layers in the rest of the macular area was substantially preserved except for a generalized granular appearance with some hyperreflective dots (Figure 6a–c). 30:2 Humphrey visual field examination highlighted an area of central scotoma in the right eye and central and nasal defects in the left eye (Figure 5d). NIR imaging showed hyper-hyporeflective dots, indicating alteration in the RPE (Figure 5c). The retinal nerve fiber layer, optic nerve head analysis, and macular ganglion cell complex thickness were preserved (Figure 7).

## 4. Discussion

SCA7 is the only spinocerebellar ataxia that seems to be associated with a decrease in visual acuity owing to retinal degeneration. In patients with SCA7, the retina develops in health before showing a progressive reduction of electroretinographic activity, retinal thinning, and silencing of genes specific to photoreceptors. CRX (cone-rod homeobox protein) dysfunction, a key transcription factor of photoreceptor genes, is central to rod and cone dystrophy. CRX requires interaction with *ATXN7* and SAGA for its transactivation activity on photoreceptor gene promoters. Additionally, dysregulation of transcriptional programs controlling the maintenance of mature photoreceptors has been found. In histological sections, SCA7 photoreceptors progressively lose their outer segments and cell polarity, acquire a round cell shape, and die via a mechanism of dark degeneration in response to mATXN7 toxicity [6,9,13]. Postmortem retinal sections reveal the almost complete loss of photoreceptors and the considerable loss of bipolar and ganglion cells resulting in severe thinning of the nuclear and plexiform layers. In addition, damage in the Bruch’s membrane, hypertrophy or degeneration of the retinal pigmentary epithelium, and optic nerve hypomyelination can be observed [2].

The ocular manifestations of SCA7 are numerous; therefore, routine ophthalmological examination is not always sufficient to assess early retinal changes that can be demonstrated with multimodal imaging. In this case series and narrative literature review, the authors present the ocular manifestations detected in three patients and analyze twenty-one publications documenting and describing the ocular manifestations detected in 151 SCA7 patients. To the best of our knowledge, these are all the case reports or case series available in English up to August 2023 that focus principally on the ophthalmological assessment and imaging methods in SCA7.

Table S1 synthesizes the articles included in the review. Numerous studies present shared characteristics of BCVA, CFP, and SDOCT as the most commonly used techniques, together with some form of color vision testing and visual field examination. Various authors reported the electrophysiological exams of patients. Less diffuse imaging techniques in the study of SCA7 are FAF, fluorescein angiography (FA), and NIR. Some studies focused on specular microscopy to analyze the morphology and cell density of the corneal endothelium. 

Central vision is compromised first and then progresses to complete blindness [3]. Visual acuity in Table S1 was converted to decimals, and the mean BCVA was 0.14 ± 0.36 in the patients reported in this study; therefore, in line with previous studies, the central vision was significantly affected. For many SCA7 patients, visual symptoms are an early manifestation of the disease, occurring before or starting together with the ataxic symptomatology. Abe et al. described a phenomenon of ophthalmologic anticipation where decreased visual acuity due to macular dysfunction started earlier in younger generations compared to older patients [14]. Around 15% of patients included in the review were younger than 18 years of age and had a visual acuity that ranged from no light perception to 0.3. 

Color perception has been reported as one of the earlier ophthalmological signs. Different studies [13,14,15,16,17,18,19] studied color perception utilizing Ishihara color tests or Farnsworth’s color test, when visual acuity permitted color testing, and described different degrees of color blindness. Four of the six patients studied by Abe et al. had tritan color blindness with loss of blue cones [14], whereas other studies indicated more aspecific color alterations. In a large case series by Velazquez et al., partial or total color blindness was commonly found in patients with adult-onset disease (above 18 years of age), while total blindness was present in nearly all patients with early onset forms (younger than 18 years of age) but in only one-third of patients with adult onset [20].

The spectrum in ocular movement anomalies is extensive, often linked to cerebellar and brainstem impairments. While many patients exhibited no discernible ocular movement disorders, the ones affected typically presented with traits such as saccadic smooth pursuit, elongated saccades, saccadic dysmetria, and eccentric gaze jerk nystagmus [13,19]. 

Funduscopic findings described by authors vary from normal–subnormal appearing maculae to Bull’s eye maculopathy and diffuse retinal atrophy. Several authors proposed a classification system for describing the fundus appearance of SCA7 patients. Campos et al. in 2000 classified retinal findings into three groups: mild retinopathy when loss of foveal reflex, moderate retinopathy with granular appearance and pigment changes of the RPE, and severe retinopathy with clinically evident atrophy [13]. More recently, in 2021, Marianelli et al. proposed a slightly different staging system composed of four stages, where CFP and SDOCT findings were classified together. Stage 0 was represented by normal CFP, with preserved foveal reflex and standard SDOCT; stage 1 showed abnormalities in macular pigmentation with a granular appearance in CFP and subfoveal cavitation on SDOCT; stage 2 was when macular atrophy was evident both on CFP and SDOCT; in stage 3, atrophic lesions were observed on the macular region, around the optic disc, and at the peripheral retina and SDOCT, it presented with diffuse photoreceptor layer atrophy [21]. This grading system is specific and easily applicable; for example, patients 1 and 3 from the case series presented herein could be classified as stage 1, whereas patient 2 would fit in stage 3. 

Other findings that authors consistently reported and that were also detected in our patients included the presence of a pale optic nerve, arterial attenuation and thinning, increased atrophy contiguous to vascular arcades, fundus hypopigmentation with generalized retinal thinning, and increasingly evident choroidal vasculature [13,14,15,19,20,21,22,23,24,25,26,27,28]. 

SDOCT is perhaps one of the most effective ophthalmological imaging methods to evaluate patients with SCA7, as it gives information on retinal thickness and a clear image of the state of the external retinal layers. In 2002, Aleman et al. were among the first to present the SDOCT characteristics of three patients with SCA7, describing foveal and parafoveal thinning with an abnormally low reflectivity splitting of the outer retina-choroidal complex [22]. Other authors, including Park et al., later confirmed these findings, describing foveal thinning with focal disruption of the ellipsoid zone and central loss of the outer segment-RPE interdigitation zone [29]. In 2021, Zou et al. described the presence of hyperreflective dots as a common finding in outer retinal and choroidal vessel layers. These hyperreflective dots seemed to correspond to the coarse granular appearance on the 633-nm scanning laser ophthalmoscope (SLO) images while remaining undetectable on CFP and with fundus autofluorescence. These authors also described that “these dots were not visible in the early stages of disease, were appreciated in an intermediate phase, and became less evident in more advanced retinopathy” [30]. It would be interesting to histopathologically study these aggregates to understand if they could represent photoreceptors that are losing polarity and undergoing dark degeneration or if perhaps they could represent mATXN7 aggregates. 

Abe et al. also utilized 633-nm SLO imaging and demonstrated a granular aspect of the posterior pole in five of the six patients they examined [14]. Loss of the ellipsoid layer, RPE changes, and decreased central retinal thickness have been reported numerous times [13,15,17,18,21,28,31,32], indicating how the central cones become affected at an early stage of the disease. In a case series including seven patients, Manrique et al. investigated the RNFL peripapillary thickness and revealed reduced thickness in all their patients, with common sparing of the temporal peripapillary quadrant. In patient 1 of our case series, similar information from the RNFL analysis seems to support this idea that the temporal fibers are less affected, in contrast to more frequent optic neuropathies that show early temporal quadrant damage [26,33,34]. 

Few authors reported FAF and FA while monitoring patients with SCA7. Zou et al. presented FAF images of three patients, two with a hypofluorescent patch in the macular area with a surrounding hyperfluorescent ring and one with more advanced retinopathy showing a dark area of macular atrophy with mottled autofluorescence in the posterior pole [30]. Ahn et al. described a bull’s eye macular configuration with FA in one patient [32]. This was not the case for a patient with foveal thinning at SDOCT, described by Park et al., where fundus examination, FA, and FAF were normal [29]. In examining patient 3 in our case series, we reported NIR images that highlighted areas of granular retinal appearance that were not visible with CFP and showed hyperreflective foveae in both eyes. NIR imaging is interesting, as its long excitation wavelength (820 nm) allows for better visualization of subretinal alterations in the photoreceptor layer, the RPE, and the choroid, compared to FAF [35].

As SCA7 patients have significant foveal disruption, visual field examinations reflect this with the presence of commonly described central scotomas [14,16,18,27,29]. Miller et al. performed visual field analysis on eight patients; only one had a normal Humphrey visual field, three had generalized depression, and four presented a central scotoma [20]. These findings are consistent with two patients described in our case series with a predominant central visual field defect. 

Electrophysiological examination is of apparent interest in understanding this pathology that affects the photoreceptors. Velázquez-Pérez et al., in 2015, conducted the most extensive case series using VEP in SCA7 patients. Documenting that impaired VEP was more frequent in patients with early-onset (71.42%) than adult-onset disease (36%). Compared with healthy controls, the most found alteration was a marked prolongation of P100 mean latency [20]. As seen in Table S1, numerous studies focused on full-field ERG, and results obtained generally demonstrated a prolonged 30-Hz flicker implicit time, with extinguished cone responses in more severely affected patients, indicating a widespread cone photoreceptor degeneration [14,15,16,17,18,19,22,25,27,28,30]. Results reported on scotopic responses were less consistent. Katagiri et al. recorded preserved rod responses in one patient [18]; Miller et al. performed full-field ERG on four patients who had affected cone responses, while only one patient showed a combination of cone and rod degeneration [19]. On other occasions, the scotopic response decreased to some degree or as much as the photopic response, suggestive of cone-rod dystrophy [11,19,22,25,27,30,31]. Multifocal ERG (mfERG), evaluating the cone function in localized areas of the macular region, recorded significantly reduced amplitudes within the foveal area; in more advanced patients, amplitudes were reduced even in the outer mfERG areas [17]. Horton et al. proposed four stages of SCA7 disease using ERG as one of the critical biomarkers. Stage 0 patients were gene positive and asymptomatic, with normal physiology (deep tendon reflexes and/or ERG); stage 1 patients had hyperreflexia or abnormal ERG; stage 2 patients had mild disease and slow progression; stage 3 patients showed rapid disease evolution [36].

Regarding endothelial cell density and corneal volume, contrasting findings were found in the literature. In 2000, Abe et al. reported that none of the six patients included in their case series presented with decreased corneal endothelial cell density. However, the method by which they reported cell density was not specified [14]. Campos et al. instead described a significantly decreased cell density and altered morphological analysis, with an inverse relationship between endothelial cell density and the number of CAG repeats [13]. In line with this, Manrique et al., using specular microscopy and Pentacam corneal topography, described a lower corneal endothelial cell density in all seven patients examined, and five also presented with an increased corneal volume [26]. 

## 5. Conclusions

SCA7, from an ophthalmological point of view, can be assessed through numerous multimodal imaging techniques to evaluate and manage the follow-up of patients. Visual acuity is a fundamental parameter that should be monitored, and SDOCT, which is a rapid and non-invasive method, should be routinely performed to evaluate ophthalmic disease progression over time. Additional exams, including NIR imaging, can indicate photoreceptor loss and disruption of the RPE that are not readily evident with CFP. Electrophysiological tests are longer examinations and more difficult for patients to undergo; however, they give essential results on the state of cone and rod dystrophy that could be paramount in guiding future genetic therapies [37,38]. 

This paper aimed to summarize the ophthalmic imaging features in SCA7 in order to increase awareness among ophthalmologists who might encounter this intricate pathology in their clinical practice. SCA7 is a rare pathology, and although only three patients are reported herein, multimodal imaging is described in detail. The limitation for future analysis derives from the fact that the number of cases reported in the literature is limited, and the majority of articles are mainly focused on the neurological aspects of the disease. Further studies on SCA7 and its ocular manifestations are needed to verify proposed classifications and possibly provide clinical guidelines for the approach to multimodal imaging in this pathology. 

## Data Availability

Not applicable.

## Supplementary Materials

The following supporting information can be downloaded at: https://www.mdpi.com/article/10.3390/life13112169/s1, Table S1: Demographic, genetic, ophthalmologic, and imaging characteristics in SCA7 patients in included studies [39].
