# Supplementary material for: Multimodal Ophthalmic Imaging in Spinocerebellar Ataxia Type 7
Source: Life (Basel). 2023 Nov 6;13(11):2169. doi: 10.3390/life13112169 (PMC10672172; doi:10.3390/life13112169)
Supplement: Supplementary file 1 [file life-13-02169-s001.zip › life-2667618-supplementary.pdf]

Supplementary Material

Table S1. Demographic, genetic, ophthalmologic, and imaging characteristics in SCA7 patients in included studies.

**Case report [Enrolled patients]**	**Pt N.**	**Age/ Sex**	**CAG rep.**	**BCVA R, L**	**Color vision R/L**	**Fundoscopy**	**SDOCT**	**Other imaging**
**Abe et al. [6]**	1<br>2<br>3<br>4<br>5<br>6	75/F<br>51/F<br>26/M<br>22/F<br>22/F<br>46/F	40<br>44<br>47<br>12<br>46<br>48	0.4 - 0.6<br>0.7 - 0.9<br>0.6 - 0.5<br>1.2 - 1.5<br>1.0 - 1.0<br>0.09 - 0.09	Tritan/tritan<br>Tritan/tritan<br>Tritan/tritan<br>N/N<br>N/N<br>Tritan/tritan	Pts 1-5: normal/subnormal fundi<br><br>Pt 6: macular atrophy, decreased pigmentation, arterial thinning, optic nerve pallor.	NP	633nn laser: coarse granular appearance in all patients except for pt 5.<br><br>Pt 6: VF with central scotomas, extinguished scotopic and photopic ERG, mERG with depression of peak response amplitude.
**Ahn et al [3]**	1<br>2<br>3	38/M<br>43/F<br>46/F	45<br>45<br>45	0.1 - 0.05<br>0.1 - 0.1<br>0.2 - 0.1	NP	Bilateral macular chorioretinal atrophy lesions	Retinal thinning that extended centrifugally outside the visibly atrophic lesions.	FA: bull's eye configuration.<br><br>mERG: reduced amplitude in a centrifugal pattern.<br><br>Microperimetry showed greater functional deficits.
**Aleman et al. [3]**	1<br>2<br>3	66/F<br>39/F<br>44/F	45<br>45<br>47	0.015 - 0.03<br>0.2 - 0.2<br>0.1 - 0.1	NP	Pt 1: Pigmentary maculopathy, temporal pallor of the optic nerve<br><br>Pts 2&3: Subtle pigment macular changes	Pts 1& 3: foveal and parafoveal thinning<br><br>Pt 2: abnormally low reflectivity splitting the outer retina-choroidal complex and parafoveal retinal thinning	Pts 1&3: reduced ERG amplitudes and delayed timing.<br><br>Pt 2: borderline rod and cone specific ERGs.
**AlHilali et al. [1]**	1	34/M	NP	0.09 - 0.1	No Ishihara color plates identified	Bull's eye maculopathy and optic disk pallor.	Loss of photoreceptor layer and foveal atrophy	Non-recordable cone ERG response.
**Atadzhanov et al. [2]**	1<br>2	20/M<br>26/F	47<br>NP	NP<br>NP	NP	1. Macular degeneration<br>2. Macular degeneration and optic nerve pallor	NP	

**Byeong-Chae et al. [1]**	1	60/M	42	0.5 - 0.33	Normal	Normal	NP	Normal VEP
**Campos-Romo et al. [16]**	12 M<br>4 F	38 ± 12.5	48.6 ± 4.6	0.04	Ishihara color test abnormal in 11 pts, normal in 1 pt and not performed in 4 pts.	2 pts had mild maculopathy (loss of foveal reflex), 4 had moderate (granular appearance and or pigment changes of the RPE), and 10 had severe (clinically evident atrophy).	Average central macular thickness 126.9 ± 17.8.<br><br>Atrophy & loss of the ellipsoid layer and RPE changes were present in 11 patients.	Specular microscopy: decreased endothelial cell density and altered morphological analysis.<br><br>ERG: decreased photopic, scotopic and combined phases.
**Gu et al. [2]**	1<br>2	7/F<br>13/F	85<br>65	CF at 30cm<br>0.13 - 0.13	NP<br>no color plates identified	1. Scattered dust-like pigmentary degeneration in the peripheral retina, normal macula, disk pallor, arterial attenuation.<br>2. Mottling of pigment at the macula, mildly scattered dust-like pigmentary degeneration in the peripheral retina, disc pallor.	NP<br>NP	Pt 2 VF: concentric narrowing.<br>Pt 2 Scotopic ERG: prolonged latency and decreased amplitude of b-waves in the right eye and absent a- and b-waves in the left eye.
**Hugosson et al. [3]**	1<br>2<br>3	21/M<br>58/F<br>34/F	47<br>38<br>34	0.5 - 0.67<br>0.1 - 0.1<br>0.17 - 0.17	aspecific color vision defects in all pts	1. Normal<br>2. Macular pigmentary changes<br>3. Normal	OCT pts 1&2: varying degrees of retinal thinning in the foveal and parafoveal areas.	mfERG: central involvement, especially in the early disease stages.<br><br>Pt 2 ERG: prolonged 30-Hz flicker implicit time.
**Italiano et al. [1]**	1	49/M	38	0.4 - 0.4	NP	Optic disk pallor bilaterally and pigmentary changes in the macular and peripheral retinal regions.	NP	VEP were impaired at low amplitudes.<br><br>Decreased amplitudes and increased latencies in photopic and scotopic ERG.
**Katagiri et al. [2]**	1<br>2	24/F<br>56/F	47-48<br>47-48	0.1 - 0.1<br>HM	Pt 1: Failure of Ishihara test	Pt 1: Atrophic macular changes	Pt 1: Foveal retinal thinning	Pt 1 perimetry: central scotomas.

			Peak n. of repeats					Pt 1ERG: reduced pattern of cone and 30-Hz flicker responses, preserved rod responses.
**Manrique et al. [7]**	1<br>2<br>3<br>4<br>5<br>6<br>7	63/F<br>29/F<br>38/M<br>65/M<br>31/M<br>46/F<br>51/M	NP	CF - CF<br>0.25 - 0.25<br>0.32 - 0.32<br>0.16 - 0.32<br>0.25 - 0.25<br>0.16 - 0.16<br>HM - HM	NP	1. Macular pseudo hole<br>2. Mild arteriolar attenuation<br>3. Arteriolar attenuation<br>4. NP<br>5. NP<br>6. Arteriolar attenuation<br>7. Arteriolar attenuation<br><br>Pigmentary changes in the macula and peripheral retina with varying degrees of optic disc pallor.	Retinal thinning in all patients.<br><br>Peripapillary RNFL thickness decreased in all patients, with common sparing of the temporal quadrants	All pts had lower corneal entothelial cell densities and 5 patients had increased corneal volume.
**Marianelli et al. [20]**	Stage 1 (9)<br><br>Stage 2 (5)<br><br>Stage 3 (6)	37.2±13.7<br><br>35.3±13.5<br><br>31±13	46±1.7<br><br>45±3.9<br><br>57.2±7.8	NP	NP	Stage 1: abnormal macular pigmentation, absence of normal foveal reflex, granular macular appearance<br><br>Stage 2: macular atrophy, pigmentary abnormalities, and different degrees of macular atrophy<br><br>Stage 3: generalized dystrophy with nummular confluent atrophic lesion, atrophy at the macula, around the optic disc and contiguous to vascular arcades.	Stage 1: abnormalities in outer retinal layers, loss of ellipsoid zone disruption of the inner segment-outer segment junction, subfoveal cavitation<br><br>Stage 2: macular atrophy, including geographic atrophy<br><br>Stage 3: diffuse atrophy of the photoreceptors layer.	
**Miller et al. [10]**	1<br>2<br>3<br>4<br>5<br>6<br>7<br>8<br>9<br>10	46/M<br>61/M<br>59/F<br>58/F<br>5/M<br>37/F<br>47/M<br>44/F<br>48/M<br>45/F	45<br>46<br>45<br>39<br>NP<br>45<br>44<br>45<br>NP<br>NP	0.29 - 0.4<br>0.05 - 0.05<br>0.1 - 0.1<br>0.4 - 0.4<br>0.05 - 0.05<br>0.2 - 0.1<br>0.1 - 0.2<br>0.005 - 0.015<br>0.1 - 0.1<br>0.5 - 0.67	Average number of correctly identified Ishihara color plates: 25% right eye and 27% left eye.	Mild granular retinal pigmented epithelium changes to bull's-eye maculopathy.	NP	ERG was performed in 4 pts, all showed reduced cone response and 1 pt had both cone and rod reduced response.

								VF: central scotomas in most patients.
**Levinson et al. [3]**	1<br>2<br>3	26/F<br>11/M<br>16/F	61<br>NP<br>NP	LP - LP<br>0.07 - 0.07<br>0.3 - 0.3	1. NP<br>2. Poor color vision<br>3. Blue-yellow deficit	1. Optic disc pallor, vascular attenuation, central macular atrophy, and peripheral pigmentary changes and bone spicules.<br>2. Optic nerve pallor and vascular attenuation.<br>3. Blunting of normal foveal reflex with granular pigmentary macular changes.	1. NP<br>2. NP<br>3. Bilateral outer retinal atrophy with loss of the subfoveal outer nuclear layer and ellipsoid zone.	1. ERG: extinguished rod and cone response bilaterally.<br>2. FA: Subtle macular changes. ERG: diminished rod and cone responses. VF: bilateral paracentral scotomas.<br>3. Normal full field ERG
**Park et al. [1]**	1	52/F	39	0.3 - 0.2	NP	Normal fundus	Foveal thinning, focal disruption of the ellipsoid zone and central loss of the outer segment-retinal pigment epithelium interdigitation zone.	FA and FAF were normal.<br><br>Fundus short wavelength autofluorescence demonstrated central weak hyper-autofluorescence in both eyes.<br><br>VF: Central scotoma<br><br>mfERG: reduction of central response.
**Pawar et al. [1]**	1	17/F	NP	0.08 - 0.08	NP	Waxy pallor of the disc with attenuated arterioles.	Foveal thinning, photoreceptor layer loss and retinal pigment epithelium irregularity.	ERG: extinguished photopic and diminished scotopic response
**Thrutell et al. [2]**	1<br>2	12/M<br>21/F	65<br>56	0.1 - 0.07<br>0.2 - 0.2	NP<br>No Ishihara plates identified	1. Pale discs, attenuated retinal arteries. Absent foveal light reflexes, subtle pigmentary mottling in the foveal region.<br>2. Normal optic disc, attenuated retinal arteries. Absent foveal light reflex, no pigmentary changes detectable.	NP	1. VF: bilateral central scotomas. mfERG: attenuated responses with absent foveal peak.<br>2. VF: bilateral central scotomas. ERG: severe cone dysfunction

								and moderate rod dysfunction.
**Velázquez-Pérez et al. [50]**	Adult onset >18 (36)<br><br>Early onset <18 (14)	NP<br><br>NP	37-46<br><br>46-72	(↓VA) 91.6%<br><br>100%	Adult onset: 39% tot., 39% color 22% partial color blindness.<br><br>Early onset: 85.71% tot., 14.28% total color blindness	Macular dysfunction 86.11%<br><br>100.00%	NP	Impaired VEP were more frequent in patients with early-onset disease (71.42%) than adult onset disease (36%)
**Wali et al. [6]**	1<br>2<br>3<br>4<br>5<br>6	22/M<br>24/M<br>54/M<br>28/F<br>20/M<br>3/M	59<br>67<br>46<br>49<br>49<br>NP	Poor<br>Blind<br>Normal<br>Normal<br>Normal<br>NP	NP	1. Pale optic discs, moderately severe pigmentary dystrophy.<br>2. Gross pallor of the optic disc and severe pigmentary dystrophy.<br>3. Normal<br>4. Normal<br>5. Normal<br>6. NP	1. Gross thinning of the fovea, thinning of the RNFL affecting the inner layers.<br>2. NP<br>3. NP<br>4. Normal retinal thickness<br>5. NP<br>6. NP	1. ERG: no recognizable wave form.<br><br>4. Normal ERG wave forms.
**Zou et al. [8]**	1<br>2<br>3<br>4<br>5<br>6<br>7<br>8	54/F<br>57/M<br>32/M<br>26/M<br>14/F<br>38/M<br>32/M<br>4/M	45<br>43<br>50<br>56<br>62<br>45<br>48<br>113	0.1 - 0.1<br>NP<br>0.4 - 0.32<br>HM - HM<br>0.1 - 0.08<br>NP<br>0.1 - 0.1<br>NLP - NLP		1. Macular atrophy<br>2. NP<br>3. NP<br>4. NP<br>5. Fundus: atrophic lesions in a petal like shape with surrounding pigment changes<br>6. NP<br>7. Macular atrophy<br>8. NP	1. Macular atrophy, hyperreflective dots<br>2. NP<br>3. NP<br>4. NP<br>5. Macular atrophy, hyperreflective dots<br>6. Normal<br>7. Macular atrophy, hyperreflective dots<br>8. NP	FAF: hypofluorescent patch in the macular area with a surrounding hyperfluorescent ring in patients 1, 5, and 7.<br><br>633-nm laser: coarse granular appearance in pts 1&3.<br><br>ERG: abnormalities tend to be more prominent in the cone-mediated.

FAF: autofluorescence; CF: counts fingers; ERG: electroretinograms; HM: hand motion; FA: fluorescein angiography; mERG: multifocal electroretinogram; NLP: no light perception; NP: not performed, SDOCT: spectral domain optical coherence tomography; Pt: patient; Pts: patients; RNFL: retinal nerve fiber layer; RPE: retinal pigment epithelium; VEP: visually evoked potentials; VF: visual field.
